# Regulation of Yujin Powder alcoholic extracts on ILC3s-TD IgA-colonic mucosal flora axis of DSS-induced ulcerative colitis

**DOI:** 10.3389/fmicb.2022.1039884

**Published:** 2022-10-20

**Authors:** Yanqiao Wen, Wangdong Zhang, Rong Yang, Lidong Jiang, Xiaosong Zhang, Baoshan Wang, Yongli Hua, Peng Ji, Ziwen Yuan, Yanming Wei, Wanling Yao

**Affiliations:** ^1^Institute of Traditional Chinese Veterinary Medicine, College of Veterinary Medicine, Gansu Agricultural University, Lanzhou, China; ^2^Laboratory of Veterinary Pathology, College of Veterinary Medicine, Gansu Agricultural University, Lanzhou, China

**Keywords:** Yujin Powder, ulcerative colitis, ILC3s, T follicular helper cells, B cells, T cell-dependent IgA, colonic mucosal flora

## Abstract

The intestinal flora maintained by the immune system plays an important role in healthy colon. However, the role of ILC3s-TD IgA-colonic mucosal flora axis in ulcerative colitis (UC) and whether it could become an innovative pathway for the treatment of UC is unknown. Yujin Powder is a classic prescription for treatment of dampness-heat type intestine disease in traditional Chinese medicine and has therapeutic effects on UC. Hence, the present study aimed to investigate the regulatory mechanism of Yujin Powder alcoholic extracts (YJP-A) on UC *via* ILC3s-TD IgA-colonic mucosal flora axis. The UC mouse model was induced by drinking 3.5% dextran sodium sulfate (DSS), meanwhile, YJP-A was given orally for prevention. During the experiment, the clinical symptoms of mice were recorded. Then the intestinal injury and inflammatory response of mice about UC were detected after the experiment. In addition, the relevant indicators of ILC3s-TD IgA-colonic mucosal flora axis were detected. The results showed that YJP-A had good therapy effects on DSS-induced mice UC: improved the symptoms, increased body weight and the length of colon, decreased the disease activity index score, ameliorated the intestinal injury, and reduced the inflammation etc. Also, YJP-A significantly increased the ILC3s proportion and the expression level of MHC II; significantly decreased the proportion of Tfh cells and B cells and the expression levels of Bcl6, IL-4, Aicda in mesenteric lymph nodes of colon in UC mice and IgA in colon. In addition, by 16S rDNA sequencing, YJP-A could restore TD IgA targets colonic mucus flora in UC mice by decreasing the relative abundance of *Mucispirillum*, *Lachnospiraceae* and increasing the relative abundance of Allprevotella, Alistipes, and Ruminococcaceae etc. In conclusion, our results demonstrated that the ILC3s-TD IgA-colonic mucosal flora axis was disordered in UC mice. YJP-A could significantly promote the proliferation of ILC3s to inhibit Tfh responses and B cells class switching through MHC II, further to limit TD IgA responses toward colonic mucosal flora. Our findings suggested that this axis may be a novel and promising strategy to prevent UC.

## Introduction

Ulcerative Colitis (UC) is a chronic, intractable, idiopathic inflammatory bowel disease (IBD), clinically mainly characterized by diarrhea and mucus, pus and blood in the stool, which mainly damages mucosa and submucosa of sigmoid colon and rectum (Ungaro et al., 2017). It diminishes the life quality and economic state for all patients (Neurath, 2019). The incidence and prevalence of UC have been continuously rising worldwide. Usually, the incidence is higher in developed country than in developing country. Although it occurs in all ages, the age onset mainly focuses on 30–40 years and there is no gender predominance (Ungaro et al., 2017; Kobayashi et al., 2020). Generally, the etiology and pathogenesis of UC are associated with genetic predisposition (Porter et al., 2020), environmental factors (Wang et al., 2010, 2017), gut microbiota (Andoh et al., 2011; Nishida et al., 2018) and dysregulated immune responses (Ungaro et al., 2017), etc. More importantly, dysfunction of intestinal mucosal barrier exerts a crucial role in the occurrence and development of UC (Li et al., 2020; Mitsialis et al., 2020). However, the precise pathogenesis is still not clear. At present, amino 5-aminosalicylic acids drugs, glucocorticoids, immunosuppressants and surgical treatment are mainly used for the treatment of UC in terms of the magnitude and extent of disease (Harbord et al., 2017; Ungaro et al., 2017; Kucharzik et al., 2020). Although UC was improved with current many therapies, even the latest drugs, the remission rate for UC is less than 50% (Kobayashi et al., 2020). Additionally, the above treatments easily lead to palindromia, drug resistance and side effects, lower immunity (de Mattos et al., 2015; Ye et al., 2020), etc. Therefore, it is an urgency to explore the pathogenesis of UC and seek innovative effective drugs.

Immunoglobulin A (IgA), the predominant antibody isotype secreted onto mucosal surfaces, is one of critical mediators of intestinal mucosal immunity barrier (Sánchez de Medina et al., 2014; Salvo Romero et al., 2015). It plays multiple immune functions in the maintenance of intestinal mucosal immune homeostasis, such as regulating the density and composition of intestinal microbiota (Peterson et al., 2007), inhibiting inflammatory reactions (Fagarasan et al., 2002), preventing the invasion of commensals and pathogens into the mucosa (Farache et al., 2013). Drops in IgA levels in the gut could increase the chances of pathogens invading into the blood circulation and stimulating immune response, while the hypersecretion of IgA could promote intestinal inflammation and induce allergy (Lamkhioued et al., 1995). Some studies demonstrated that the expression of IgA levels in ileum (Rabah et al., 2020) and colon (Liu et al., 2019) was significantly increased in DSS-induced mice UC. It is now known that there are two regulatory pathways of IgA generation, including T cell-dependent (TD) and T cell-independent (TI) pathways. In particular, TD pathway results in the generation of high-affinity and single-reaction IgA, which targets various pathogens (Cerutti, 2008). Group 3 Innate lymphoid cells (ILC3s) play multiple regulatory roles in the maintenance of intestinal immune homeostasis. In particular, LTi-like ILC3s located in colonic lymphatic tissues (Montaldo et al., 2015) is not only involved in the formation (Lee et al., 2011) and repair (Scandella et al., 2008) of gut-associated lymphatic tissues (GALT), but, more importantly, the activation of LTi-like ILC3s will interact with T follicular helper (Tfh) cells, and the responses of commensal microbiota specific IgA and pathogenic bacteria specific IgA were reduced by limiting Tfh cells response and B cells class switch *via* antigen presentation, further regulated colonic mucosal flora (Melo-Gonzalez et al., 2019). Therefore, ILC3s-TD IgA-colonic mucosal flora axis exerts a central role in the regulation of intestinal mucosal immune homeostasis. However, the immune modulatory mechanisms of this axis in UC intestinal mucosal injury have not been reported.

Many studies and long-term clinical practice have proved that herbs have certain advantages in treatment of UC (Li et al., 2019; Liu et al., 2020; Qiao et al., 2020). Complementary and Alternative Medicine (CAM) based on Traditional Chinese Medicine (TCM) is currently used by over 50% of IBD patients (Holleran et al., 2020). According to the theory of TCM, many scholars believe that the invasion of large intestine by dampness and heat is the root of UC (Qing, 2017; Sun et al., 2017). Yujin Powder (YJP) is a classic prescription for the treatment of large intestine damp-heat diarrhea. Our previous studies have shown that YJP had treatment effect on large intestine dampness-heat syndrome by anti-diarrhea, anti-inflammation, repairing intestinal mucosa, regulating the balance of oxidation and antioxidation, adjusting gastrointestinal hormone (Wen et al., 2017; Yao et al., 2017; Zhang et al., 2018; Yao et al., 2019). And modern pharmacological studies have also shown that the active ingredients of YJP have the effects of anti-inflammation, repairing intestinal mucosa and anti-ulcer (Gautam et al., 2013; Kant et al., 2014; Ji et al., 2019; Song et al., 2020). Ruiqiong Wang et al. found that the semi-bionic extracts of YJP had protective effects on acute enteritis induced by Dextran Sulfate Sodium (DSS) in rats (Wang et al., 2016). Thus, in this study, we aimed to investigate the role of ILC3s-TD IgA-colonic mucosal flora axis in the pathogenesis of UC and the regulatory effects of YJP-A on this axis.

## Materials and methods

### Experimental animals

Healthy male BABL/c mice (6–8 weeks old, 18–20 g) were provided by the Animal Center of the Lanzhou Veterinary Research Institute of the Chinese Academy of Agricultural Sciences [SCXK (Gan) 2020-0002]. The mice were acclimatized for 1 week before experiment and given free access to forage and water *ad libitum* in the condition of 12 h light–dark cycle with temperature and humidity ranging from 18 to 25°C and 50 ± 5%. Animal welfare and experimental procedures were performed in strict accordance with the *Guidelines for the Management and Use of Laboratory Animals* (Ministry of Science and Technology of China, 2006) and approved by the Animal Ethics Committee of Gansu Agricultural University and the Animal Protection and Utilization Committee.

### Drugs and reagents

The herbs for YJP were purchased from Yellow River Chinese Medicine Co. Ltd. (Lanzhou, Gansu, China) and were authenticated by Prof. Yanming Wei in the College of Veterinary Medicine of Gansu Agricultural University. YJP is composed of eight herbal medicines (Table 1). The Voucher specimens of the herbs were deposited in Traditional Chinese Veterinary Medicine Laboratory of Gansu Agricultural University. Reference standards: curcumin (CAS: 458-37-7,), gallic acid (CAS: 149-91-7), chezic acid (CAS: 18942-26-2), geniposide (CAS: 24512-63-8), paeoniflorin (CAS: 23180-57-6), coptisine (CAS: 6020-18-4), berberine (CAS: 2086-83-1), baicalin (CAS: 21967-41-9), baicalein (CAS: 491-67-8), wogonoside (CAS: 51059-44-0), wogonin (CAS: 632-85-9), emodin (CAS: 518-82-1) and chrysophanol (CAS: 481-74-3) were supplied by Nanjing Yuanzhi Biotechnology Co., Ltd. (Nanjing, China). All these standards were purchased with purities higher than 98%. Dextran sulfate sodium (DSS; MW: 36000–50,000) was provided by MP Biomedicals, LLC (Solon, OH, United States). Sulfasalazine (SASP) was purchased from Shanghai Xinyi Tianping Pharmaceutical Co., Ltd. (Shanghai, China). Anhydrous ethanol was obtained from Tianjin Baishi Chemical Plant Co., Ltd. (Tianjin, China).

**Table 1:** Description of Yujin Powder (YJP).

Medicinal plant	Voucher specimen number	Ratio	Origin (China)
Curcumae Radix (*Curcuma kwangsiensis* S. G. Lee et C. F. Liang., radix)	GSAU-CVM-20150018	2	Guangxi province
Chebulae Fructus (*Terminalia chebula* Retz., fruit)	GSAU-CVM-20150019	1	Yunnan provinc
Scutellariae Radix (*Scutellaria baicalensis* Georgi., radix)	GSAU-CVM-20150020	2	Hebei provinc
Rhei Radix Et Rhizoma (*Rheum palmatum* L., radix and rhizome)	GSAU-CVM-20150021	4	Gansu province
Coptidis Rhizoma (*Coptis chinensis* Franch., rhizome)	GSAU-CVM-20150022	2	Sichuan province
Gardeniae Fructus (*Gardenia jasminoides* Ellis., fruit)	GSAU-CVM-20150023	2	Zhejiang province
Paeoniae Radix Alba (*Paeonia lactiflora* Pall., radix)	GSAU-CVM-20150024	1	Hebei province
Phellodendri Chinensis Cortex (*Phellodendron chinense* Schneid., cortex)	GSAU-CVM-20150025	2	Sichuan province

Anti-MHC II antibody (CAS: bs-4107R), anti-IgA antibody (CAS: bs-0774R) were obtained from Beijing Biosynthesis Biotechnology Co., Ltd. (Beijing, China). anti-Bcl6 antibody (CAS: sc-7388) was purchased from Santa Cruz Biotechnology, Inc. (Santa Cruz, CA, United States). anti-β-Tublin antibody (CAS: 66240-1-Ig), anti-GAPDH antibody (CAS: 60004-1-Ig), HRP-conjugated Affinipure Goat Anti-Mouse or Anti-Rabbit IgG (CAS: SA00001-1 and SA00001-2) were purchased from Proteintech Group, Inc. (Wuhan, China). And Goat anti-Rabbit IgG H&L (Alexa Fluor 488; CAS: ab150077) was obtained from Abcam Plc, Inc. (MA, United States). FITC anti-CD45 (CAS: 553080), BUV 395 anti-B220 (CAS: 563793), PE anti-CD19 (CAS: 557399), BUV 395 anti-CD3 (CAS: 565992), PE anti-CD4 (CAS: 553730), APC anti-CXCR5 (CAS: 560615), PE anti-CD127 (CAS: 552543), Alexa Fluor 647 anti-RORγt (CAS: 563620) were purchased from BD Biosciences (New Jersey, United States). eFluor 660 anti-GL7 (CAS: 50–5,902-82) was purchased from Thermo Fisher Scientific (MA, United States).

### Preparation YJP-A

Curcumae Radix, Coptidis Rhizoma, Scutellariae Radix, Phellodendri Chinensis Cortex, Gardeniae Fructus, Rhei Radix Et Rhizoma, Paeoniae Radix Alba, Chebulae Fructus were crushed, sifted through the 60 mesh screen and mixed in a ratio of 2:2:2:2:2:4:1:1. The mixture was performed by ultrasound and then extracted by the 60% ethanol (solid–liquid ratio: 1:25, 50°C, 50 min) refluxing for twice. The filtrate obtained twice was mixed and evaporated by rotary evaporator at 60°C and then freeze-dried by vacuum freeze dryer. The dry extract was accurately weighed and obtained yield of 31%.

### Quality control analysis of YJP-A

To control the quality of YJP-A, the main active ingredients were detected by high-performance liquid chromatography (HPLC) using the Agilent 1,260 Infinity LC system (Agilent Technologies, USA). The column configuration consisted of an Agilent Zorbax SB-C18 column (4.6 mm × 250 mm, 5 μm). The mobile phrases were, respectively, composed of (A) aqueous phosphoric acid (0.1%, v/v) and (C) acetonitrile (gallic acid, chezic acid, geniposide, paeoniflorin, coptisine, berberine, baicalin, baicalein, wogonoside, wogonin), (A) aqueous acetic acid (10%, v/v) and (C) acetonitrile (curcumin, emodin and chrysophanol) at the flow rate of 1.0 ml/min. The gradient elution was optimized as follows: 1–2 min, 95–85% A; 2–13 min, 85–80% A; 13–20 min, 80–62% A; 20–30 min, 62–52% A; 30–35 min, 52–41% A (gallic acid, chezic acid, geniposide, paeoniflorin, coptisine, berberine, baicalin, baicalein, wogonoside, wogonin); 1–2 min, 95–85% A; 2–10 min, 85–70% A; 10–30 min, 70–40% A; 30–40 min, 40–40% A (curcumin, emodin and chrysophanol). The detection wavelengths were, respectively, set at 250 and 425 nm. The column temperature was maintained at 25°C, and injection volume was 20 μl.

### UC model establishment and YJP-A intervention

**Figure 1:**
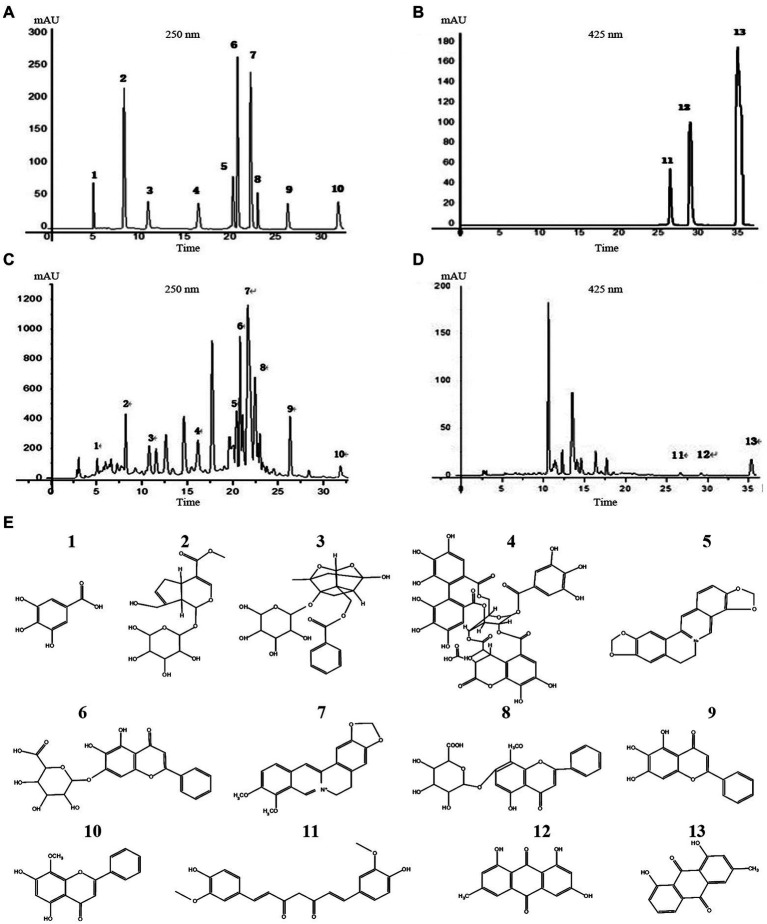
YJP-A ameliorates DSS-induced mice UC. **(A)** Experiments design. Body weight change **(B)** and disease activity index **(C)** of the mice during experiment period. YJP-A alleviates DSS-induced colon length reduction **(D,E)**. Symptoms of hematochezia and diarrhea in mice of each group **(F)**. Data are expressed as the mean ± SEM, *n* = 8 per group. ^###^*p* < 0.001 vs. NC group; ***p < 0.001 vs. model group.

The animal experiment progress is shown in Figure 1A. The BABL/c mice were randomly divided into normal control (NC), Model, SASP (0.45 g/kg), YJP-A high dose (YJP-A-H; 14.56 g/kg, weight ratio between crude drug and mice), YJP-A middle dose (YJP-A-M; 7.28 g/kg) and YJP-A low dose (YJP-A-L; 3.64 g/kg) groups with 8 mice in each group. Except NC group was given distilled water, other groups were given 3.5% DSS drinking water for 7 consecutive days. At the same time, each intervention group mice were orally given different dose of YJP-A and SASP dissolved in 0.5% carboxymethylcellulose sodium (CMC-Na) solution gavaged with corresponding drug twice a day, (12 h intervals), and corresponding volume of 0.5% CMC-Na solution in NC and Model groups. The volume of intragastric administration was 0.2 ml/10 g body weight.

### Clinical observation and assessment of disease activity index

The changes of mental and active state, hairs, appetites, weight, stool consistency, occult blood/thick blood of stool were observed and recorded daily during the experiment. And DAI of each mice was monitored according to the Cooper method (Cooper et al., 1993) with more detail as follows: (a) body weight loss: 0 points = none; 1 points = 1–5% loss; 2 points = 5–10% loss; 3 points = 10–20% loss; 4 points = over 20% loss. (b) diarrhea: 0 points = normal; 1 point = soft but still formed; 2 points = soft; 3 points = very soft and wet; 4 points = watery diarrhea. (c) hematochezia: 0 points = negative hemoccult; 1 point = weakly positive hemoccult; 2 points = positive hemoccult; 3 points = blood traces in stool visible; 4 points = gross rectal bleeding. On day 8, the mice were sacrificed and the first node of mesenteric lymph nodes (MLNs) chain, proximal to the cecum and the colorectum were collected and the colon length from the anus to ileocecal junction were measured and recorded.

### Histopathological analysis

The colon tissue was fixed with 10% neutral formalin, embedded in paraffin and cut into 3-μm-thick sections for haematoxylin and eosin (HE) staining. Pathological photographs were captured by Olympus microphotography system. Histopathological score was used to quantify the degree of colon tissue injury, including the degree of intestinal epithelial cell injury and the inflammatory infiltration. The scoring criteria were outlined as follows (Ding and Wen, 2018): 0 (normal morphology and no inflammation), 1 (a fraction of goblet cell loss and mild inflammatory infiltration), 2 (large area of goblet cell loss and moderate inflammatory infiltration), 3 (small amounts or partial crypt loss and extensive inflammatory infiltration of the mucosal muscular layer with mucosal edema and thickening), 4 (large area of crypt loss and extensive inflammatory infiltration of submucosa layer).

### ELISA detection

The colonic tissues and MLNs of mice were homogenized and the supernatant were collected. Then, the myeloperoxidase (MPO) activity in colonic tissue was detected according to the instructions of MPO assay kit (Nanjing jiancheng, Nanjing, China). And the cytokines contents of TNF-α, IL-6, IL-1β, IL-10 and IgA in colonic tissue and IL-4, Aicda in MLN were measured by ELISA according to the instructions of mouse ELISA kit (Neobioscience, Shenzhen, and Mlbio, Shanghai, China), respectively. The total protein concentration in homogenates was determined by BCA protein assay kit (Solarbio, Beijing, China) to normalize the cytokines concentration.

### Flow cytometry assay

MLNs were removed from mice under aseptic conditions and the single cell suspension was obtained by 70 U cell strainers. Then single cell suspension was stained by adding different antibodies as follow: anti-CD45-FITC, anti-B220-BUV 395, anti-CD19-PE, anti-GL7-eFluor 660, anti-CD3-BUV 395, anti-CD4-PE, anti-CXCR5-APC, anti-CD127-PE. For the nuclear marker RORγt, the cell membrane was broken by Foxp3 nuclear transcription factor breaking staining solution (Thermo Fisher, MA, United States) and then added to anti-RORγt-Alexa Fluor 647. Finally, Flow cytometry analyses were performed on CytoFLEX LX 5L19C (Beckman Coulter Inc., United States).

### Western blot assay

The MLNs were cut into pieces and lysed in RIPA buffer (Solarbio, Beijing, China) with protein phosphatase inhibitor (Solarbio), and total proteins were extracted according to the manufacturer’s protocols. Then, the protein concentration was detected by BCA protein assay kit (Solarbio). Each protein sample (50 μg) was electrophoresed on separating 10% SDS-polyacrylamide gel electrophoresis (SDS-PAGE) and then transferred to 0.22 μm polyvinylidene fluoride (PVDF) membrane (Millipore, MA, United States) at 220 mA for 1.5 h. Afterwards, PVDF membrane was sealed with 5% non-fat powdered milk for 2 h at room temperature and incubated with anti-MHC II antibody (1:500) and anti-Bcl6 (1:250) overnight at 4°C, respectively. The next day, PVDF membrane was washed with 1 × Tris–HCl-buffered saline Tween-20 (TBST) five times for 5 min and incubated with secondary antibodies: HRP-conjugated Affinipure Goat Anti-Mouse or Anti-Rabbit IgG (1,5,000) for 1.5 h at room temperature. Finally, the PVDF membrane was washed and imaged using Amersham Imager 600 chemiluminometer (GE Healthcare Bio-Sciences AB, Sweden). The gray values of protein bands were analyzed by ImageJ software.

### Immunofluorescence assay

Three μm thick paraffin sections of colonic tissues were dewaxed with xylene and gradiently alcohol solution, tissue antigen retrieval was performed with sodium citrate solution (120°C for 10 min). The sections were washed with phosphate buffered saline (PBS, PH = 7.4) solution and sealed with 5% BSA for 1 h at room temperature. Then the primary antibody (IgA, 1:400 dilution) was incubated overnight at 4°C. The sections were washed with PBS solution after returning to room temperature and then incubated with Goat Anti-Rabbit IgG (Alexa Fluor 488 Conjugate, 1:400) in the dark at 37°C for 1 h. Finally, the sections were washed with PBS solution again and anti-fluorescence attenuating tablet (with DAPI; Solarbio, Beijing, China) was added to seal. And then the sections were observed and the photographs were collected using a confocal laser scanning microscope (Carl Zeiss, Germany).

### Molecular docking

The molecular structures of MHC II (AlphaFold ID: P33076), Bcl6 (PDB ID: 6XMX), IL-4 (PDB ID: 1HIJ), Aicda (PDB ID: 5W0R) and active ingredients (gallic acid, gardenoside, paeoniflorin, paeoniac acid, coptisine, baicalin, berberine, baicalin, baicalin, baicalin, curcumin, emodin, chrysophanol) were constructed and preprocessed using Autodock 4.4.6. According to the interaction between the active ingredients and macromolecular proteins, the central location, length, width and height of the Grid Box were determined. Then, molecular docking was performed using AutoDock 4.4.6 and the binding free energy was calculated by Lamarckian. Finally, the docking results were visualized using PyMOL 2.2.0 to analyze the interaction between the main active ingredients in YJP-A and the key proteins.

## Colonic mucosal microflora assay

### Total DNA extraction and Illumina Miseq sequencing of colonic mucosal flora

The colons were cut longitudinally along the mesentery side and washed with PBS solution to remove intestinal contents. Then the intestinal mucus was gently scraped 5 times with sterile slides and collected into the eppendorf tubes. Total bacterial DNA was extracted from colonic mucus of mice according to the TGuide S96 Soil/fecal DNA Isolation kit (Tiangen, Beijing, China) instructions. The concentration and purity of DNA were detected by agarose gel electrophoresis and UV spectrophotometer. Then, the V3-V4 hypervariable region of 16S rDNA gene was amplified by PCR using primers 338F (5′- ACTCCTACGGGAGGCAGCA-3′) and 806R (5’-GGACTACHVGGGTWTCTAAT-3′). Subsequently, the amplified products of PCR were purified and quantified by Ampure XP magnetic beads and Nanodrop 2000, respectively. And then the sequencing library was homogenized according to the mass ratio of 1:1. Finally, the qualified libraries were sequenced using Illumina Novaseq 6,000.

### Preprocessing and analysis of sequencing data

The sequencing data (raw reads) was filtered by Trimmomatic v0.33 to obtain clean reads without primer sequences. Usearch V10 was used to splice through overlapping relations, and length filtering was performed on the spliced data according to length range of different regions. Finally, UCHIME V4.2 was used to identify and remove chimeric sequences to obtain effective reads. The effective reads were clustered under 97% similarity and the representative OTU (Operational Taxonomic Units) sequence were obtained through Usearch V 10. Subsequently, SILVA was used as the reference database to carry out taxonomic annotation on the feature sequence with Navie Bayesian Classifier, and the corresponding species classification information to each feature were obtained. Further, QIIME V1.80 was used to perform Alpha and Beta diversity analysis to reflect species richness and diversity of samples, compare similarity of species diversity among different samples, The plots of principal component analysis (PCA) and partial least squares discriminant analysis (PLS-DA) were performed based on R language platform. Finally, potential microbial biomarkers in each group were identified by LEfSe analysis.

### Statistical analysis

Statistical analysis was performed with SPSS version 26.0 (IBM, Armonk, NY, United States). All data were presented as mean ± standard error of mean (SEM). Statistical analysis was performed using one-way analysis of variance (ANOVA) with LSD analysis. All the figures were plotted using GraphPad Prism version 6.0 (GraphPad Software Inc., La Jolla, CA, United States). Statistical significance was set at *p <* 0.05.

## Results

### Quality control of YJP-A

**Figure 2:**
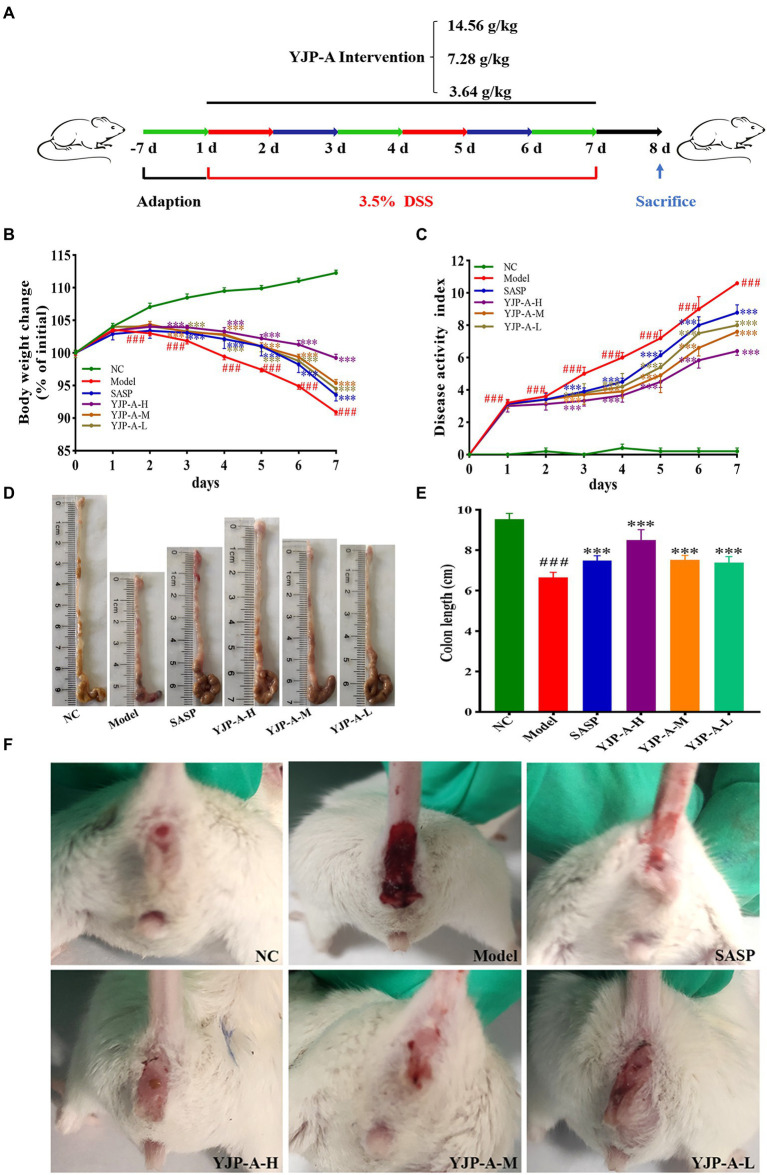
HPLC chromatogram of standard solutions **(A,B)**, YJP-A sample solutions **(C,D)**. The structural formula of chemical compounds in YJP-A **(E)**: 1. gallic acid; 2. gardenoside; 3. paeoniflorin; 4. chebulinic acid; 5. coptisine; 6. baicalin; 7. berberine; 8. wogonoside; 9. baicalein; 10. wogonin; 11. curcumin; 12. emodin; 13. chrysophanol.

Thirteen main active ingredients in YJP-A were detected by HPLC (Figure 2). The concentration of thirteen main active ingredients-gallic acid, gardenoside, paeoniflorin, chebulinic acid, coptisine, baicalin, berberine, wogonoside, baicalein, wogonin, curcumin, emodin, chrysophanol in YJP-A, were 17.99 ± 0.10, 98.33 ± 0.21, 188.60 ± 0.20, 160.60 ± 0.69, 26.70 ± 0.10, 118.27 ± 0.3, 111.47 ± 0.06, 22.50 ± 0.52, 31.80 ± 0.03, 11.80 ± 0.01, 1.50 ± 0.01, 41.80 ± 0.02, 0.05 ± 0.003 mg/g, respectively.

### YJP-A ameliorates DSS-induced mice UC

During the animal experiment, the mice showed lethargy, depression, curled up, tanglesome hairs and dietary wishes reducing. YJP-A (especially YJP-A-H) could improve the above symptoms. The body weight decreased significantly on the second day (*p* < 0.001; Figure 1B). On day 3, the stools were soft. The bloody stool was even observed on the fourth day. The DAI of DSS-induced mice increased significantly every day (*p* < 0.001; Figure 1C). After establishing model, strikingly, the UC mice exhibited colon shortening (*p* < 0.001; Figures 1D,E). YJP-A intervention (especially YJP-A-H) showed good protective effects on UC mice, referred to the facts of better mental status, body weight gaining (Figure 1B), bloody stool alleviating (Figure 1F), colon lengthening (Figures 1D,E), as well as a lower DAI score (*p* < 0.001; Figure 1C). Results above-described indicated that the UC mice model was successful established; meanwhile, YJP-A had good protective effects on UC, and the YJP-A-H had the best protective effects, followed by the YJP-A-M and YJP-A-L.

### YJP-A ameliorates intestinal injury and inflammation response of UC mice

**Figure 3:**
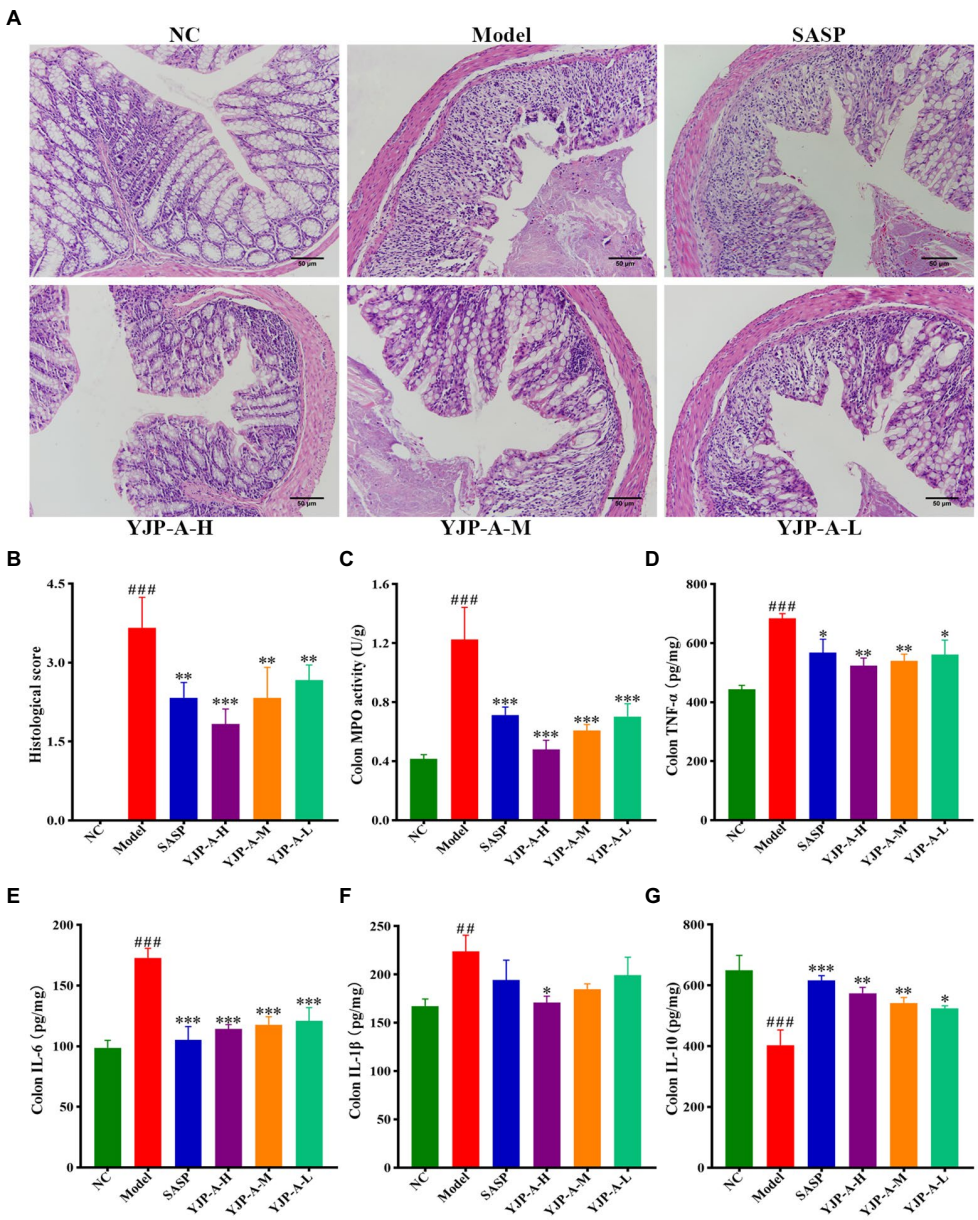
YJP-A ameliorates intestinal injury and inflammation response in UC mice. **(A)** Histopathology changes of each group in colon (200×). **(B)** Histological scores of colons. **(C)** MPO activity in colon. **(D–G)** Changes of colonic inflammatory cytokines levels, including TNF-α **(D)**, IL-6 **(E)**, IL-1β **(F)** and IL-10 **(G)**. Data are expressed as the mean ± SEM, except for histopathological analysis *n* = 3 per group, the remaining analysis *n* = 5 per group. ^##^*p* < 0.01, ^###^*p* < 0.001 vs. NC group; *p < 0.05, **p < 0.001, *** *p* < 0.0001 vs. model group.

The intestinal epithelium of DSS-induced UC mice was severely damaged and exfoliated, there were many inflammatory cells infiltration, edema and hyperemia in the mucosa and submucosa and the structure of intestinal crypt and villi was blurred (Figure 3A). Therefore, compared with the NC group, the histopathological score of colonic tissue in the model group increased significantly (*p* < 0.001; Figure 3B). Different dose of YJP-A could significantly improve the damage and inflammation, including mucosa exfoliation, inflammatory cells infiltration and crypt loss etc., moreover, the effect of the YJP-A-H was the best (Figure 3A).

In addition, MPO activity of colonic tissue in UC mice was significantly increased (*p* < 0.001; Figure 3C). However, YJP-A (especially YJP-A-H) could significantly reduce MPO activity of colonic tissues in UC mice (*p* < 0.001 or *p* < 0.01; Figure 3C). The levels of TNF-α, IL-6 and IL-1β in colon were significantly increased (*p* < 0.001 or *p* < 0.01; Figures 3D–F), while the levels of IL-10 were significantly decreased in UC mice (*p* < 0.001; Figure 3G). YJP-A could reduce the levels of TNF-α and IL-6 (*p* < 0.001, *p* < 0.01 or *p* < 0.05), increase the levels of IL-10 (*p* < 0.01 or *p* < 0.05), and YJP-A-H could significantly reduce the levels of IL-1β in colonic tissue (*p* < 0.05; Figures 3D–G). These results suggested that there were significant inflammatory responses in UC mice. YJP-A could significantly alleviate the inflammation of colonic tissue in UC mice. The regulation effect of the YJP-A-H was the best, followed by the YJP-A-M and YJP-A-L.

### YJP-A up-regulates ILC3s proportion and expression of MHC II in MLNs of UC mice

**Figure 4:**
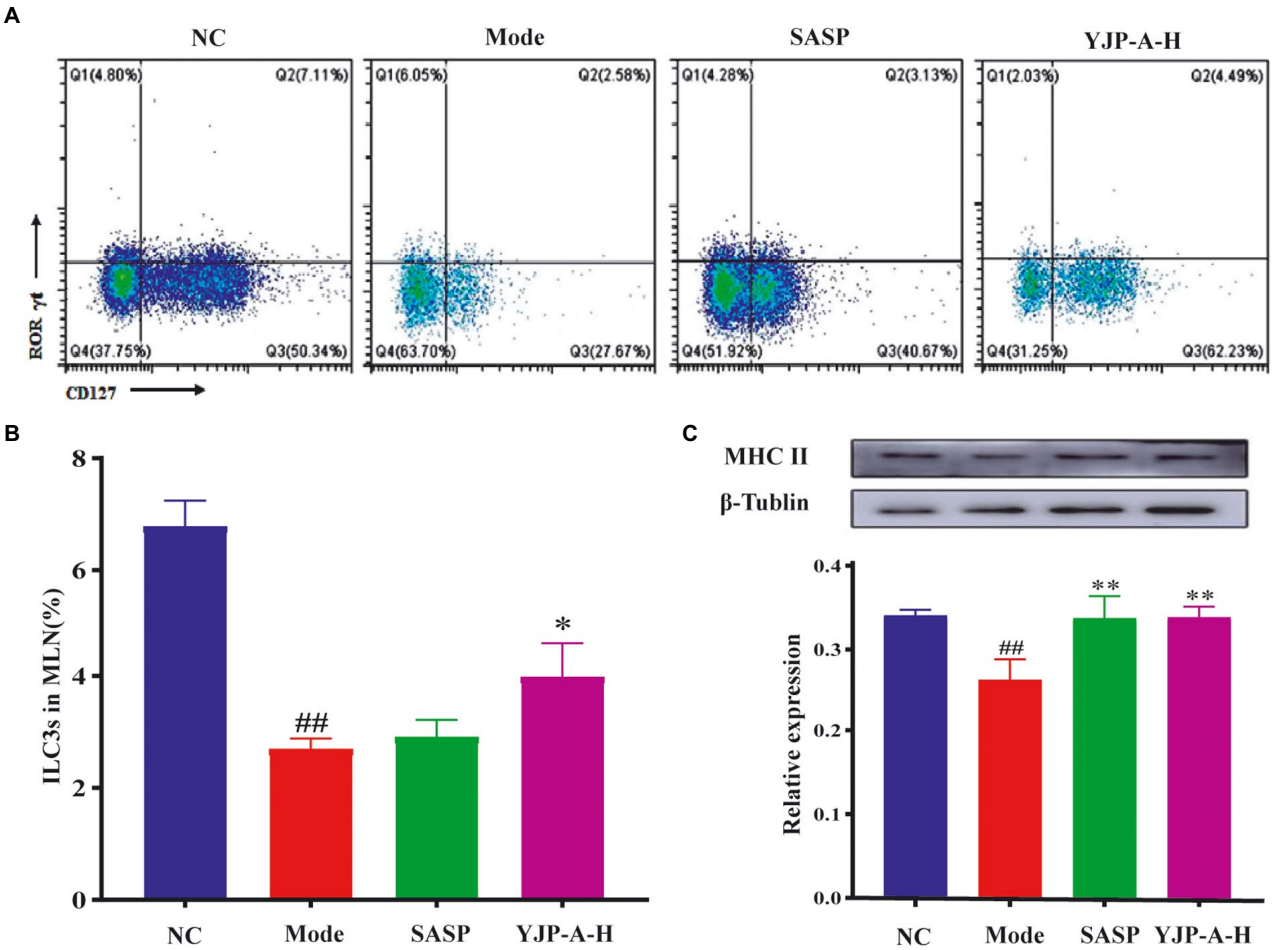
Effects of YJP-A-H on changes of ILC3s **(A, B)**, and MHC II expression levels **(C)** in MLNs. Data are expressed as the mean ± SEM, except for flow cytometry assay *n* = 3 per group, the remaining analysis *n* = 5 per group. ^##^*p* < 0.01, vs. NC group; *p < 0.05, **p < 0.01 vs. model group.

The pharmacodynamic experiment proved that high dose of YJP-A has the best preventive effect in UC mice. Therefore, we used to high dose of YJP-A for subsequent experiments. The changes of ILC3s and MHC II expression in MLNs were detected by flow cytometry and Western Blot. The proportion of ILC3s and the expression of MHC II in MLNs of UC mice were significantly decreased (*p* < 0.01; Figure 4). YJP-A-H could significantly up-regulate the proportion of ILC3s and the expression of MHC II (*p* < 0.05 or *p* < 0.01; Figure 4).

### Inhibition effect of YJP-A on Tfh cells in MLNs of UC mice

**Figure 5:**
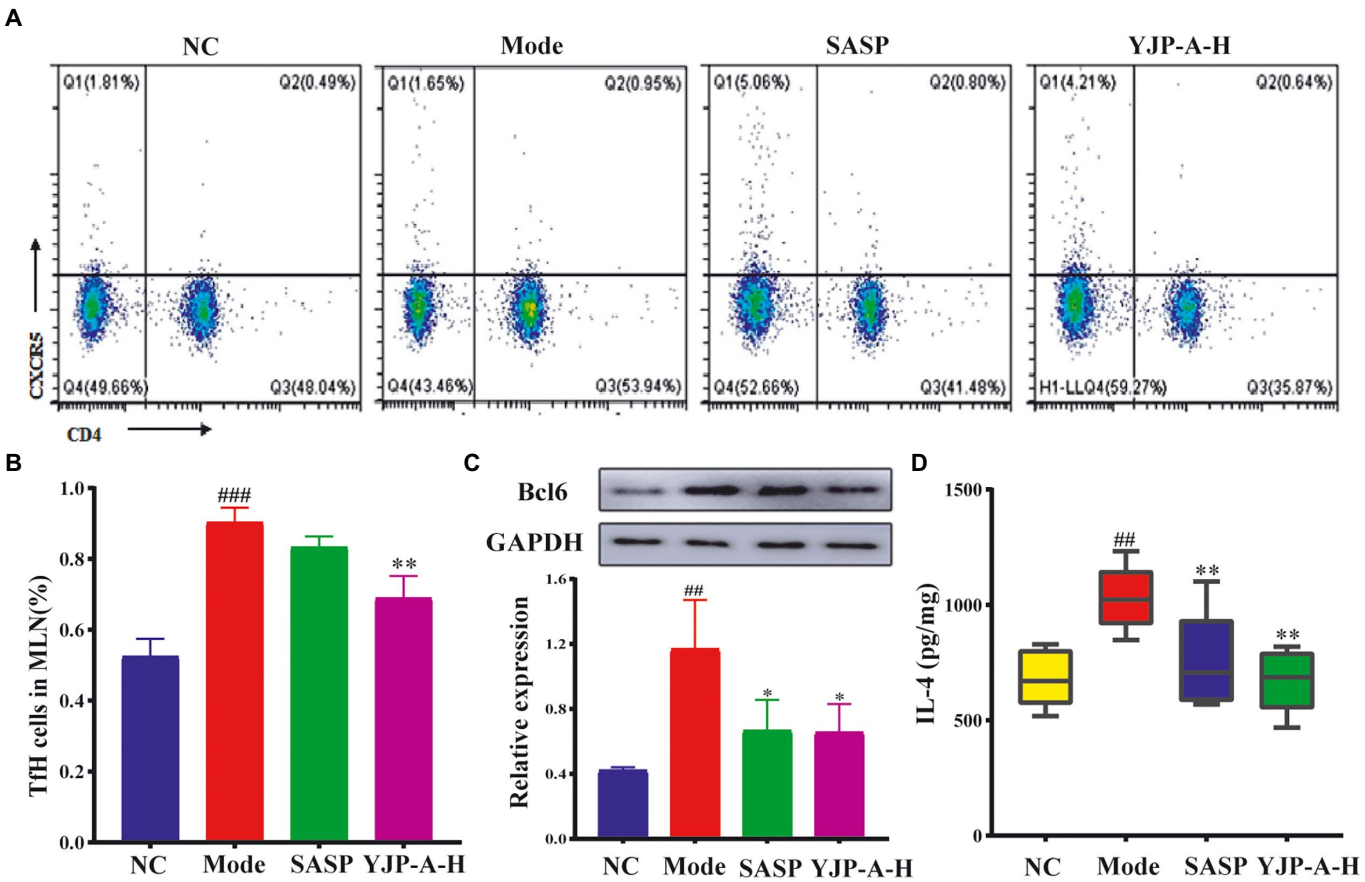
Effects of YJP-A-H on changes of Tfh cells **(A,B)**, the expression of Bcl6 **(C)** and IL-4 **(D)** in MLNs. Data are expressed as the mean ± SEM, except for flow cytometry assay *n* = 3 per group, the remaining analysis *n* = 5 per group. ^##^*p* < 0.01, ^###^*p* < 0.001 vs. NC group; *p < 0.05, **p < 0.01 vs. model group.

The changes of Tfh cells and Bcl6 and IL-4 expression were detected. The proportion of Tfh cells and Bcl6 and IL-4 expression levels were significantly increased in MLNs of UC mice (*p* < 0.001 or *p* < 0.01; Figure 5). YJP-A-H intervention could significantly down-regulate the proportion of Tfh cells and Bcl6 and IL-4 expression levels in MLNs (*p* < 0.05 or *p* < 0.01; Figure 5).

### Upregulation of YJP-A on B cells in MLNs and mucosal IgA

**Figure 6:**
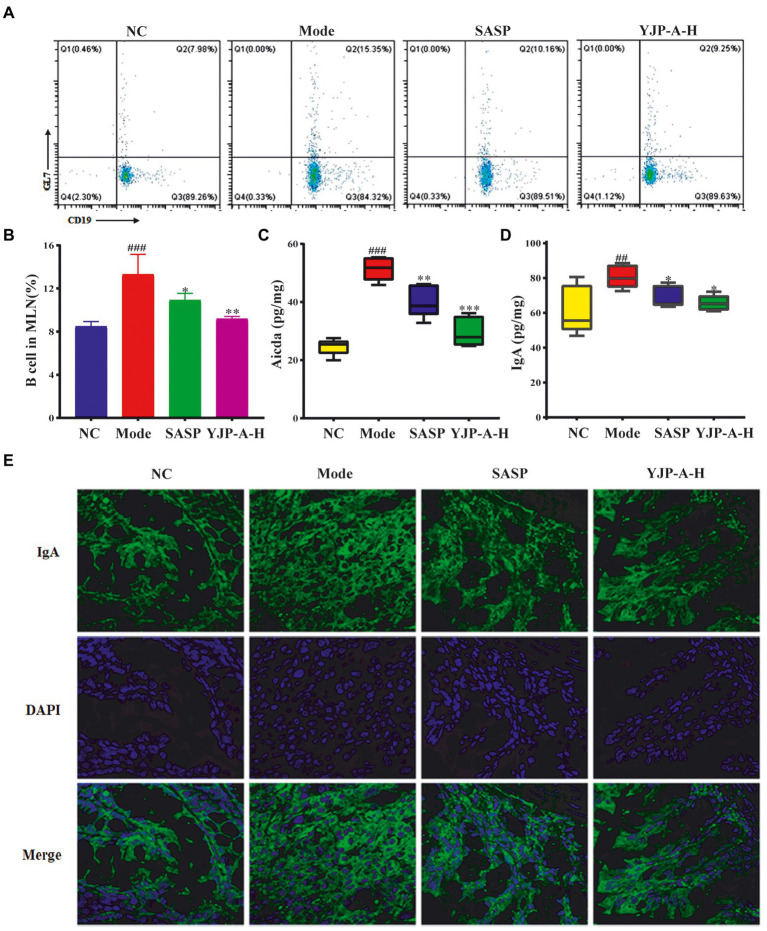
Effects of YJP-A-H on changes of B cells **(A,B)**, Aicda **(C)** in MLNs and IgA levels **(D,E)** in colon. Data are expressed as the mean ± SEM, except for flow cytometry assay *n* = 3 per group, the remaining analysis *n* = 5 per group. ^##^*p* < 0.01, ^###^*p* < 0.001 vs. NC group; *p < 0.05, **p < 0.01, ***p < 0.001 vs. model group.

Based on the above results, we tested the changes of B cells and Aicda expression in MLNs and mucosal IgA. The proportion of B cells and Aicda expression levels in MLNs and IgA on colonic mucosa were significantly increased in UC mice (*p* < 0.001 or *p* < 0.01; Figure 6). YJP-A-H could significantly decrease the proportion of B cells and Aicda expression in MLNs, and then down-regulate the IgA levels on colonic mucosa (*p* < 0.001, *p* < 0.01or *p* < 0.05; Figure 6).

### Molecular docking

**Figure 7:**
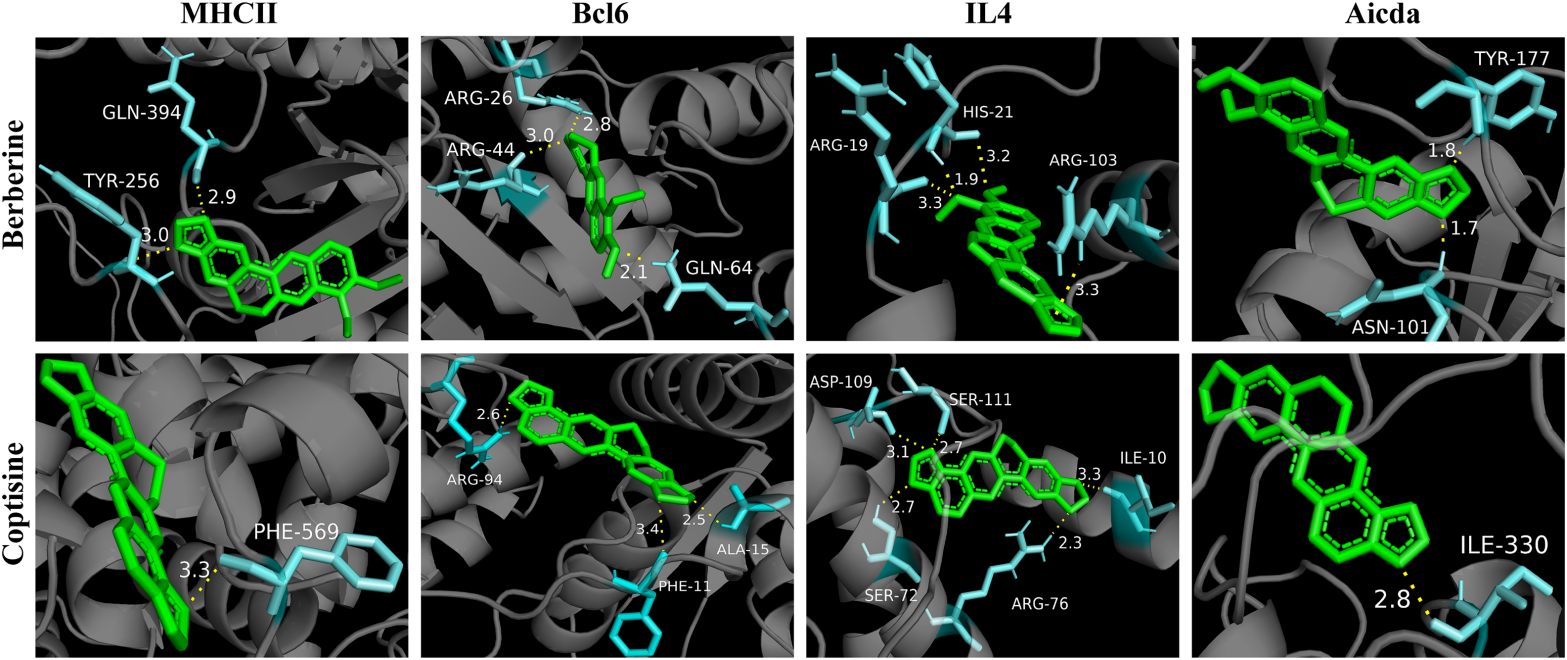
Molecular docking diagram of berberine and coptisine in YJP-A and the key proteins (MHC II, Bcl6, IL-4, Aicda).

Furtherly, in order to verify the regulation of YJP-A on ILC3s-TD IgA-colonic mucosal flora axis, the molecular docking technology was performed to research the interaction of 13 active ingredients in YJP-A and the key proteins (MHC II, Bcl6, IL-4, Aicda) in the above signal pathway. The results showed that binding energy of main active ingredients in YJP-A with the above key proteins was strong, especially berberine and coptisine (Table 2; Figure 7), indicating that these active ingredients could activate ILC3s-TD IgA-colonic mucosal flora axis by regulating the above proteins.

**Table 2:** Molecular docking free energy.

Active ingredients	Binding free energy (KJ·mol^−1^)			
	MHC II	Bcl6	IL-4	Aicda
Curcumin	−3.27	−3.19	−6.44	−3.60
Gallic acid	−2.89	−3.96	−5.00	−4.96
Chebulinic acid	−3.27	−4.76	−7.99	−4.37
Gardenoside	−1.49	−0.73	−3.04	−2.93
Paeoniflorin	−1.77	−1.36	−5.84	−2.28
Coptisine	−6.94	−5.94	−7.27	−5.88
Berberine	−6.69	−6.04	−7.60	−6.77
Baicalin	−2.43	−3.64	−5.56	−2.51
Baicalein	−4.28	−4.61	−6.02	−4.99
Wogonoside	−3.05	−4.66	−6.80	−4.51
Wogonin	−4.84	−4.06	−5.88	−5.07
Emodin	−5.02	−4.68	−6.88	−5.48
Chrysophanol	−5.33	−5.09	−6.46	−5.48

## YJP-A regulates TD IgA targeted colonic mucosal flora of UC mice

### Effects of YJP-A on the diversity and abundance of colonic mucosal flora in UC mice

**Figure 8:**
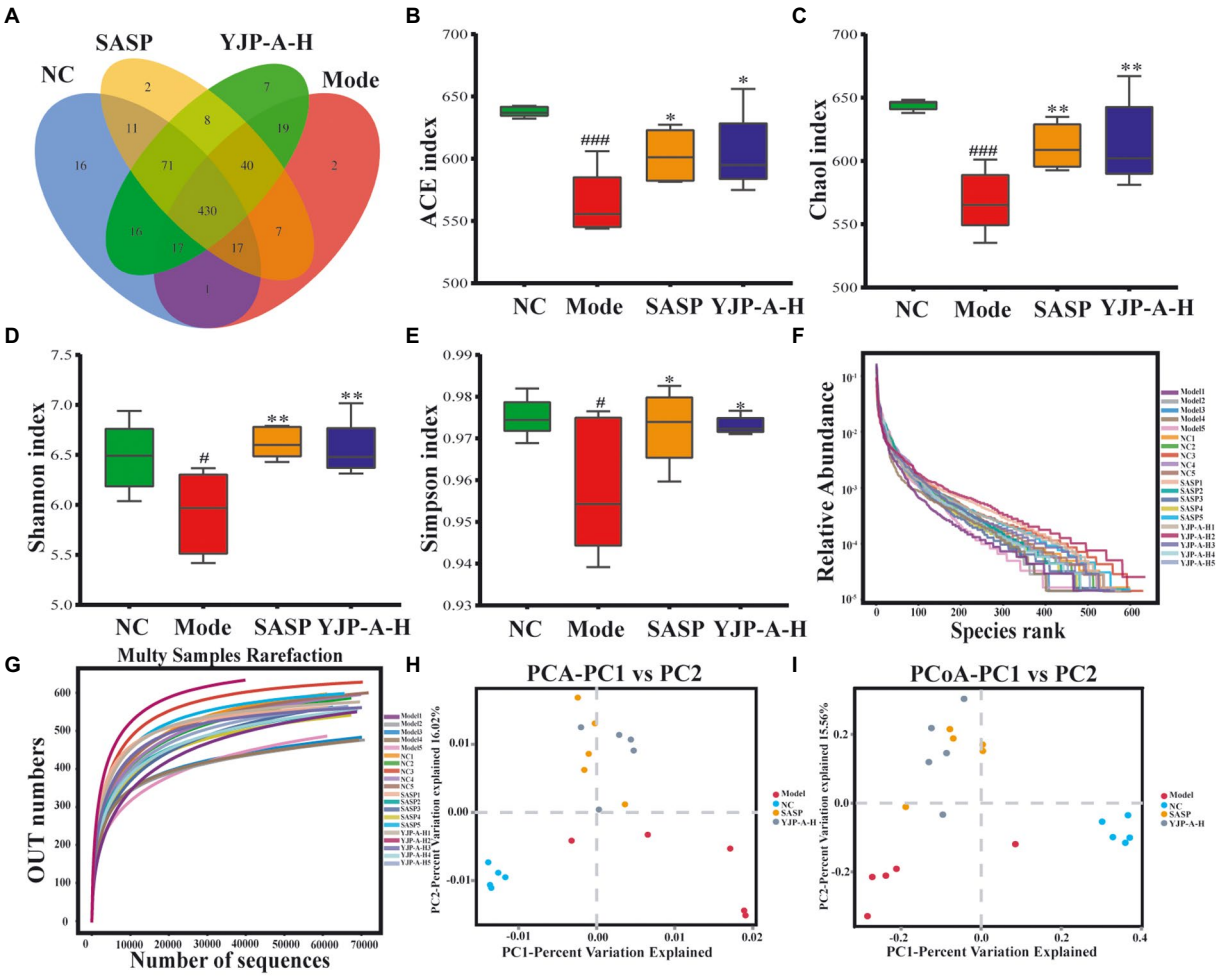
Effects of YJP-A-H on colonic mucosal flora diversity and abundance of UC mice. **(A)** Species Venn diagram analysis. **(B)** ACE, **(C)** Chao1, **(D)** Shannon and **(E)** Simpson indices. Rank abundance curve **(F)** and rarefaction curve **(G)**. PCA plot **(H)** and PCoA plot **(I)** of colonic mucosal flora. Data are expressed as the mean ± SEM, *n* = 5 per group. ^#^*p* < 0.05, ^###^*p* < 0.001 vs. NC group; *p < 0.05, **p < 0.01 vs. model group.

TD IgA responses are regulated by the flora colonized in colonic mucosa. Therefore, in order to further explore the effects of YJP-A on colonic mucosal flora of UC mice, the 16S rDNA of colonic mucosal flora was sequenced and analyzed. According to the Venn diagram (Figure 8A), in total there were 430 OTUs in four groups, among which there were 16, 2, 2 and 7 unique OTUs in the NC group, Model group, SASP group and YJP-A-H group, respectively. Based on OTU, alpha diversity analysis was performed. The results showed that the ACE, Chao1, Shannon and Simpson indices were all significantly decreased in UC mice (*p* < 0.001 or *p* < 0.05; Figures 8B–E). YJP-A-H could significantly increase the above indices (*p* < 0.01 or *p* < 0.05; Figures 8B–E). The results indicated that the abundance and evenness of colonic mucosal flora in UC mice were significantly decreased, and YJP-A-H had significant regulatory effects. In addition, rank abundance curve was flat (Figure 8F), indicating that species composition of all samples had high uniformity. Meanwhile, the rarefaction curve showed that the curve of each sample tended to be flat with the increase of sequence numbers (Figure 8G), indicating that the data volume of sequencing was sufficient to reflect the species diversity. These results further suggested that the reduction in alpha diversity in UC mice was not caused by sequencing process. Based on these results, principal component analysis (PCA) and principal coordinate analysis (PCoA) were performed. The flora of the NC and Model groups were clearly separated (Figures 8H,I). While YJP-A-H group deviated from the NC and Model groups and formed another flora. These results demonstrated that the composition of colonic mucosal flora in UC mice changed significantly. YJP-A-H significantly changed the colonic mucosal flora profile of UC mice.

### Effects of YJP-A on relative abundance of colonic mucosal flora in UC mice at the phylum and genus levels

**Figure 9:**
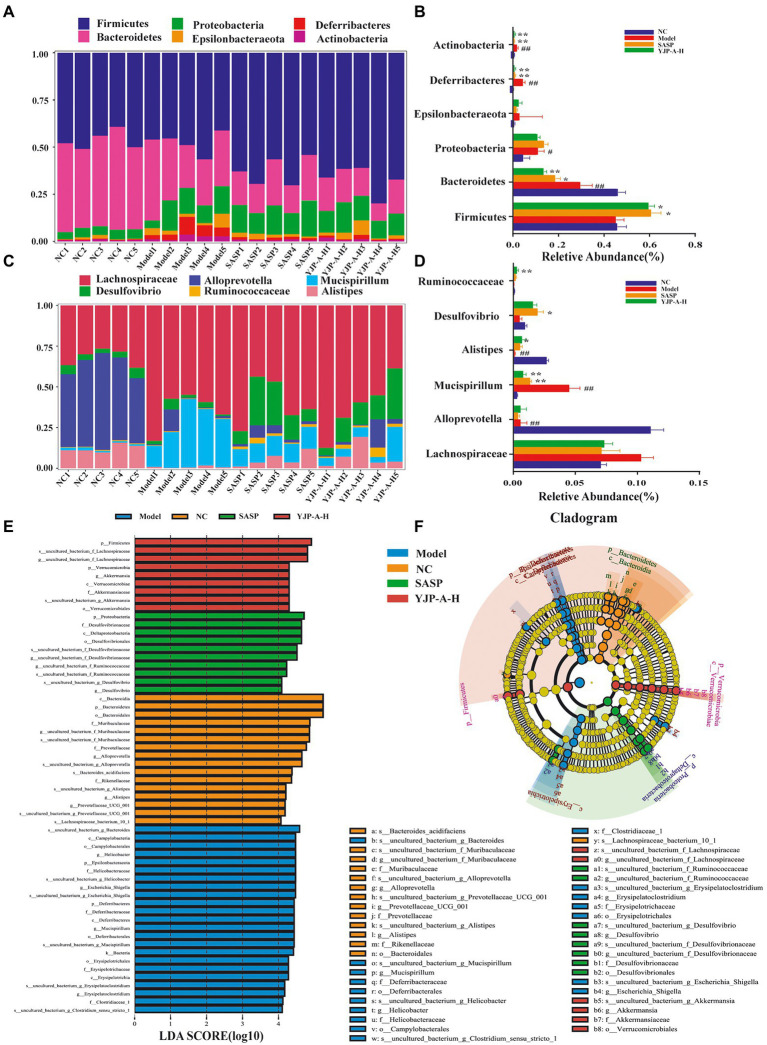
Effects of YJP-A-H on colonic mucosal flora structure in UC mice. Difference analysis at phylum **(A,B)** and genus **(C,D)** levels in mice of each group. **(E)** LDA score distribution histogram. LDA score > 4.0 indicates differentially abundant genera which was considered as biomarkers. **(F)** LEfSe cladogram. The root of the cladogram denotes the domain bacteria. Different colors indicate the different group hosting the greatest abundance. The size of each node represents their relative abundance. Data are expressed as the mean ± SEM, *n* = 5 per group. ^#^*p* < 0.05, ^##^*p* < 0.01 vs. NC group; *p < 0.05, **p < 0.01 vs. model group.

In view of composition and abundance of colonic mucosal flora changed significantly, difference significance test among groups was performed at phylum and genus levels by ANOVA to explore the regulation of YJP-A on UC mice. The results showed that Firmicutes, Bacteroidetes and Proteobacteria were dominated at the phylum level (Figures 9A,B). Specifically, the relative abundance of Bacteroidetes significantly was decreased (*p* < 0.01), and Firmicutes was decreased, but there was no significant difference (*p* > 0.05), while others (e.g., Proteobacteria, Actinobacteria, Deferribacteres, Epsilonbacteraeota) were significantly increased in UC mice (*p* < 0.01), but YJP-A-H could normalize this phenomenon besides Bacteroidetes. At the genus level (Figure 9C), the relative abundance of *Mucispirillum* (*p* < 0.01) and *Lachnospiraceae* (*p* > 0.05) were significantly and no significantly increased; nevertheless, the relative abundance of *Allprevotella* and *Alistipes* were significantly decreased (*p* < 0.01), and the relative abundance of *Desulfovibrio* and *Ruminococcaceae* were lowered (*p* > 0.05) in UC mice. YJP-A-H restored the ratios of these bacterial genera to normal levels. These results revealed that the structure of colonic mucosal flora was significantly perturbed, showing that pathogens increased while probiotic decreased significantly. YJP-A-H could effectively regulate the disorder, meanwhile, it is also indicated that YJP-A-H have certain antibacterial effects.

The biometric biomarkers among groups were further indentified by LEfSe, and 58 biomarkers were found (LDA score log^10^ > 4; Figure 9E). Cladogram showed that the biomarkers in NC group were *Allprevotella*, *Alistipes*, *Prevotellaccace* and *Muribaculaceae*; in Model group were *Helicobacter*, *Mucispirillum*, *Escherichia* and *Erysipelatoclostridium*; in YJP-A-H group were *Lachnospiraceae* and *Akkermansia* at genus level (Figure 9F).

### Correlation analysis

**Figure 10:**
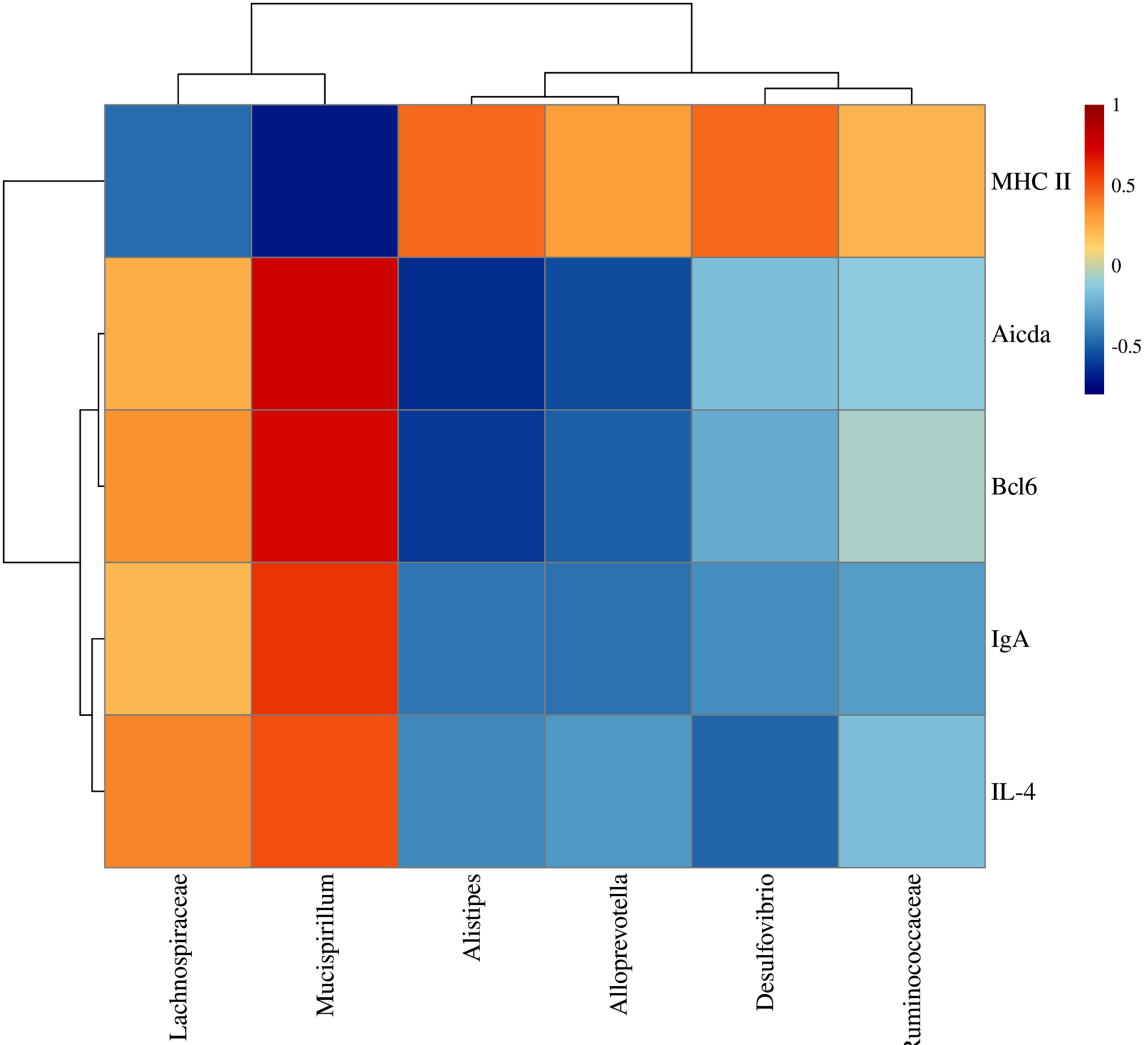
Correlation analysis between TD IgA related indexes and colonic mucosal flora of UC mice after YJP-A treatment.

Furtherly, to illustrate the potential relationship between the colonic mucosal flora and TD IgA reaction, we performed correlation analysis by Pearson correlation analysis. MHC II was positively correlated with *Allprevotella*, *Alistipes*, *Desulfovibrio*, *Ruminococcaceae* and negatively with *Mucispirillum* and *Lachnospiraceae* (Figure 10). On the contrary, Bcl6, IL-4, Aicda and IgA were positively related to *Mucispirillum*, *Lachnospiraceae* and negatively related to *Allprevotella*, *Alistipes*, *Desulfovibrio* and *Ruminococcaceae* (Figure 10).

## Discussion

UC is an obstinate inflammatory disease of colonic mucosa, and it is urgent need to find new drugs for treatment. YJP is a classic prescription for the treatment of the large intestine dampness-heat diarrhea, which is often used to diarrhoeal disease of animals caused by bacterial, viral and parasitic infection, etc. And the semi-bionic extracts of YJP had protective effect on experimental colitis (Wang et al., 2016). In the present study, it was proved that YJP-A had protective effects on UC mice by alleviating weight loss, diarrhea, bloody stools, colon shortening, intestinal injury and inflammation (Figures 1, 3). Recently, most studies on the pathogenesis of UC focus on the abnormal mucosal immune response (Zundler et al., 2019). Therefore, we explored the role of ILC3s-TD IgA-colonic mucosal flora axis in the pathogenesis of UC and the regulation of YJP-A.

ILC3s, “communication center” in intestinal mucosal immunity homeostasis, have ability to perceive a variety of exogenous and endogenous signals (Geremia and Arancibia-Cárcamo, 2017). Other studies have reported that the changes of ILC3s were detected in intestinal mucosa and peripheral blood of UC and Crohn patients or experimental animals (Chang et al., 2021; Kong et al., 2021; Li et al., 2021; Zhou et al., 2021). However, whether ILC3s in MLNs changed is unknown. Interestingly, in the present study, we firstly found that the proportion of ILC3s and MHC II level expressed by ILC3s in MLNs of UC mice were also significantly decreased. YJP-A strikingly increased the ILC3s proportion and dramatically enhanced MHC II expression (Figure 4; Hepworth et al., 2015). It is indicated that ILC3s and the expression of MHC II were strongly limited in MLNs of UC mice and YJP-A could relieve this limit.

Emerging research has demonstrated that ILC3s in MLNs of mice could inhibit Tfh cells and their functions *via* MHC II (Melo-Gonzalez et al., 2019). Therefore, followingly, we detected the amount of Tfh cells and main associated factor in MLNs of UC mice. Strikingly, we observed the increased Tfh cells ratio in MLNs of UC mice. A banch of studies have confirmed that producing specific antibodies is the most effective way for humoral immunity to prevent and defend against pathogens (Locci et al., 2016). Tfh cells belong to CD4^+^T cell subset. The well-differentiated Tfh cells play an important role in germinal center (GC) formation, affinity maturation, and the development of most high-affinity antibodies and memory B cells through producing cytokines, costimulatory molecules and a series of inhibitory receptor (e.g., IL-21, IL-4, CXCL13, ICOS, PD-1 and BTLA etc). It is worth mentioning that, although Tfh cells differentiation is a multi-stage and multi-factor process, the transcription factor-Bcl6 is one of the most regulatory factors in driving its differentiation (Nurieva et al., 2009; Yu et al., 2009). Correspondingly, a significant increase of Bcl6 expression in MLNs of UC mice was appeared. Therefore, combined with the findings of Felipe Melo-Gonzalez et al. that ILC3s in MLNs of mice could inhibit Tfh cells *via* MHC II (Melo-Gonzalez et al., 2019), it is strongly demonstrated that ILC3s and ILC3s-expressed MHC II were dramatically limited in the MLNs of DSS-induced UC mice correspondingly reduced the inhibition on Tfh cells. We found that YJP-A could enhance the inhibition of ILC3s on Tfh cells (Figures 5A–C).

The extensive researches showed that IgA had absolute superiority in adaptive mucosal immune response, while IL-5, IL-10 and IL-21 could accelerate the differentiation of B cells towards plasma cells secreting IgA in MLNs (Bagheri et al., 2019; Hashiguchi et al., 2020). Recently, other studies have proven that the Tfh cells could also mediate class switching of IgA^+^ B cells through expressed IL-4 (Melo-Gonzalez et al., 2019). Combined with the above research results, we speculated that the expression of IL-4 in MLNs would rise, further B cells increase Aicda expression-indicative of somatic hypermutation and class switch recombination in MLNs, and promote IgA expression levels in colon. Thus, in the present study, the expression level of IL-4 in MLNs of UC mice was detected. We found a significant increase of IL-4 expression (Figure 5D). Followingly, we detected the proportion of B cells, Aicda expression in MLNs and IgA levels in colon. We found that they all significantly increased (Figure 6). These results were completely congruent with the above prediction. And these results were reversed following YJP-A intervention (Figures 5D, 6). It demonstrated that proliferated Tfh cells induced the class switch of B cells toward IgA^+^ cells *via* IL-4 in MLNs of UC mice, which further led to the elevated GC responses and IgA production. YJP-A could improve this phenomenon.

IgA is the main effector molecule of intestinal mucosal immune defense barrier and plays a crucial role in the maintenance of intestinal mucosal immune homeostasis and mutualism with the mucosal-dwelling commensal microbiota (Pabst and Slack, 2020). Hyperfunction of TD IgA response could result in an increase of single-reaction and high-affinity IgA, which led to disorder of some commensal bacteria colonized on mucus layer or the intestinal epithelium and increase of IgA-coated bacteria species (Melo-Gonzalez et al., 2019). The proliferation and invasion of a large number of pathogenic bacteria will destroy mucous layer and increase permeability, further leading to the damage of intestinal mucosal epithelial cells and inducing inflammatory response of the mucosal immune to cause colitis (Ng et al., 2013; Li et al., 2015). In the present study, strikingly, a lower diversity and abundance and changed flora structure of colonic mucosa flora in UC mice were found. YJP-A had good improvement (Figure 8). Some studies have shown that the relative abundance of Lachnospiraceae was significantly increased in DSS-induced UC of mice (Wei, 2018), which was closely related to colon mucosal TD IgA (Melo-Gonzalez et al., 2019). An increase of relative abundance of Lachnospiraceae in colonic mucosa of UC mice were observed in the present study (Figure 9D), indicating that this might be due to TD IgA hypersecretion. Additionally, we found that relative abundance of Mucispirillum was also increased (Figure 9D). Some studies have shown that Mucispirillum, as unique microflora of colonic mucus, could trigger TD IgA response through antigen presentation (Robertson et al., 2005). In addition, the rise of relative abundance of Mucispirillum is closely associated with the occurrence of IBD (Song et al., 2018). While the relative abundance of classic SCFAs-producing bacteria Allprevotella, Alistipes and Ruminococcaceae were all decreased in UC mice in the present study (Figure 9D). Consistently, some researches also showed that the relative abundance of SCFAs-producing bacteria were reduced in UC mice (Zhu et al., 2020; Hu et al., 2021a). Furthermore, there is literature to indicate that the enhancement of TD IgA response resulted in the reduction of relative abundance of partial SCFAs-producing bacteria (Melo-Gonzalez et al., 2019). Thus, the above results demonstrated that TD IgA response hypersecretion in UC mice could induce disorder of colonic mucosal flora, especially TD IgA targeted flora, further aggravated the injury of colon mucosa. We found that YJP-A had good regulation (Figure 9D).

**Figure 11:**
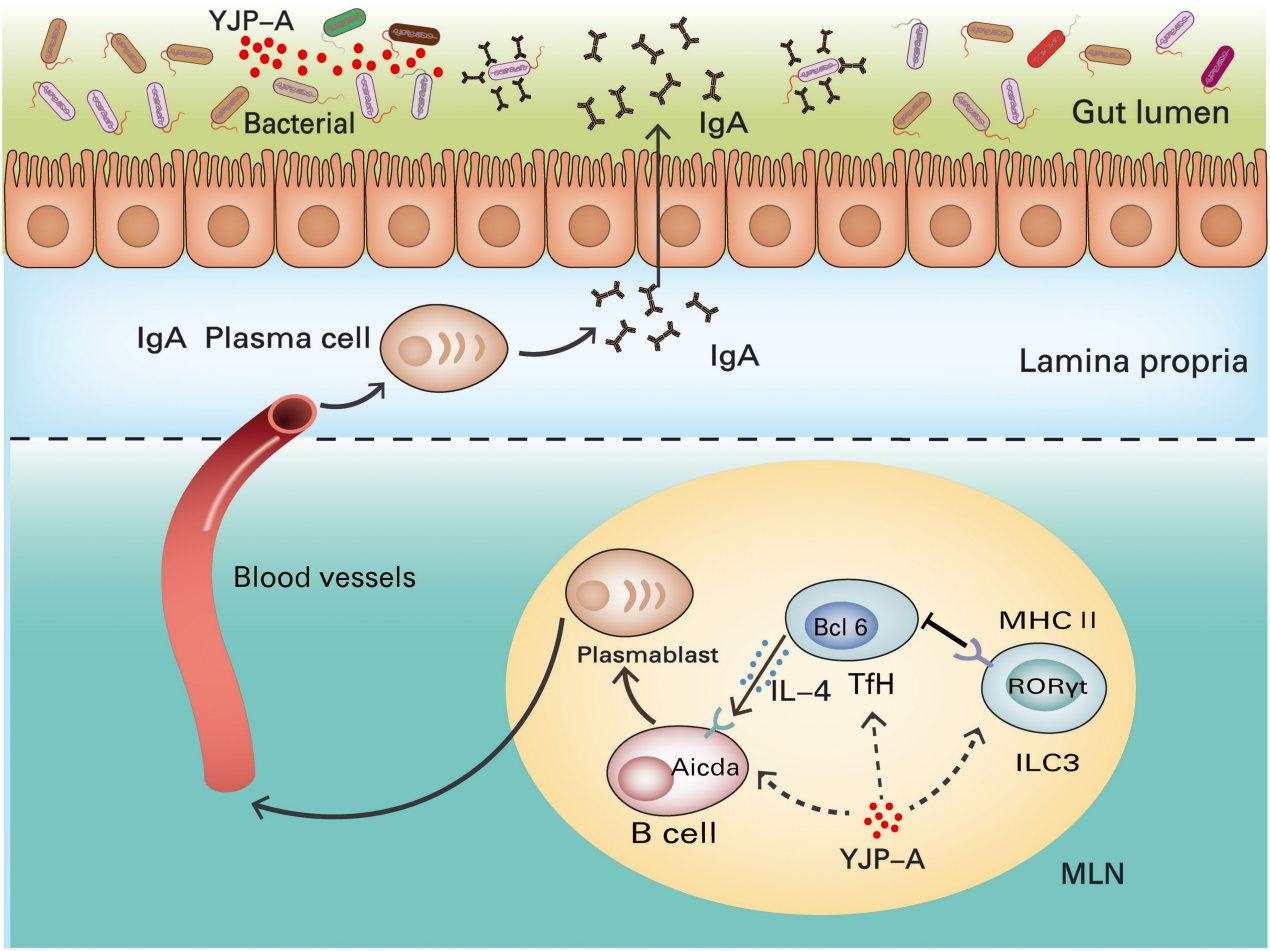
Mechanisms of YJP-A ameliorating DSS-induced ulcerative colitis by ILC3s-TD IgA-colonic mucosal flora axis. The ILC3s and MHC II expression were suppressed in MLNs of DSS-induced UC mice, resulting in the proliferation of Tfh cells and inducing class switching of IgA^+^ B cells by increasing IL-4 levels and hypersecretion of TD IgA in turn causing the disorder of colonic mucosal flora. YJP-A showed protective effects on UC mice by regulating ILC3s-TD IgA-colonic mucosal flora axis.

In summary, YJP-A exerted anti-UC effects on DSS-induced mice UC through upregulating the proportion of ILC3s and expression of MHC II, enhancing the inhibition of ILC3s on Tfh cells in MLNs, further regulating TD IgA response and TD IgA targeted colonic mucosal flora. Further, the molecular docking verified the strong interaction between 13 active ingredients in YJP-A and the crucial macromolecular proteins including MHC II, Bcl6, IL-4, Aicda in the ILC3s-TD IgA-colonic mucosal flora axis (Table 2; Figure 7). Some studies showed that YJP-A and (or) its main active ingredients have potential regulatory effects on some points in ILC3s-TD IgA-colonic mucosal flora axis. For instance, our previous studies have shown that YJP could reduce IgA levels in serum (Zhang et al., 2018) and the expression of ileal and colonic mucosal SIgA in Large Intestine Dampness-heat Syndrome (Yao et al., 2019); berberine, the active component of *Coptidis Rhizoma* and *Phellodendri Chinensis Cortex*, and paeoniflorin, a main ingredient of *Paeoniae Radix Alba*, could enhance immune function by lowering IgA levels (Li et al., 2012; Tang et al., 2021); baicalin, a main ingredient of *Scutellaria baicalensis*, regulates intestinal flora through promoting the production of SCFAs and further protects dysfunction of intestinal mucosal immune (Hu et al., 2021b); baicalein ameliorates UC by improving intestinal epithelial barrier *via* AhR/IL-22 pathway in ILC3s (Li et al., 2021); Huangqin decoction exerted therapeutic effect on UC mice by increasing the proportion of ILC3s and MHC II expression to suppress the function of Th17 and Th1 cells and promote Treg and Th2 cells (Zhou et al., 2021); curcumin and paeoniflorin could ameliorate UC by regulating gut microbiota (Huang et al., 2017; Fan et al., 2020). Therefore, the combination of these medicines, YJP-A could ameliorate DSS-induced UC in mice by regulating ILC3s-TD IgA-colonic mucosal flora axis (Figure 11).

## Conclusion

Taken together, we evaluated the role of ILC3s-TD IgA-colonic mucosal flora axis in the pathogenesis of UC and the regulation of YJP-A on the above axis. Our study illustrated that the ILC3s and MHC II expression in MLNs of UC mice were strongly suppressed and its inhibitory effect on Tfh cells was reduced, leading to the proliferation of Tfh cells and induced the class switching of IgA^+^ B cells by increasing IL-4 levels, and hypersecretion of TD IgA caused the disorder of colonic mucosal flora, further aggravated colon mucosa. YJP-A could ameliorate UC through regulating the ILC3s-TD IgA-colonic mucosal flora axis with a characteristic of multiple-components and targets. It is demonstrated that ILC3s-TD IgA-colonic mucosal flora axis is an innovative pathway of immune regulation of colonic mucosal inflammation. It plays a crucial role in the occurrence and development of UC. The present study broadened the strategy of treatment of UC and found new pathway of YJP-A treating disease. This provides a new and powerful evidence for exploring the pathogenesis of UC and new drug development.

## Data availability statement

The datasets presented in this study can be found in online repositories. The names of the repository/repositories and accession number(s) can be found in the article/Supplementarymaterial.

## Ethics statement

The animal study was reviewed and approved by Animal Ethics Committee and the Animal Protection and Utilization Committee of Gansu Agricultural University.

## Author contributions

YW designed, performed the experiment, and drafted the manuscript. WY designed the study, formulated the experimental scheme, interpreted the data, and co-drafted the manuscript. WZ participated in the design of the study, the formulation of the experimental scheme, and the revision of the manuscript. RY, LJ, XZ, and BW participated in experimental operation. YH, PJ, and ZY participated in data processing. YW contributed to the design of the study, the interpretation of data, the revision, and the approval of the final manuscript. All authors contributed to the article and approved the submitted version.

## Funding

This research was supported by the Natural Science Foundation of China (grant nos. 31802231 and 32060812); initial funding for Scientific Research of Gansu Agricultural University (2017RCZX-14); China Agriculture Research System-37 (CARS-37) and the supporting foundation of Gansu province College industry (2020C-14).

## Conflict of interest

The authors declare that the research was conducted in the absence of any commercial or financial relationships that could be construed as a potential conflict of interest.

## Publisher’s note

All claims expressed in this article are solely those of the authors and do not necessarily represent those of their affiliated organizations, or those of the publisher, the editors and the reviewers. Any product that may be evaluated in this article, or claim that may be made by its manufacturer, is not guaranteed or endorsed by the publisher.

## Abbreviations

UC: Ulcerative colitis
IBD: Inflammatory bowel disease
IgA: Immunoglobulin A
TD IgA: T cell-dependent IgA
ILC3s: Group 3 Innate lymphoid cells
Tfh: T follicular helper
GALT: Gut-associated lymphatic tissues
CAM: Complementary and Alternative Medicine
TCM: Traditional Chinese Medicine
DSS: Dextran Sulfate Sodium
YJP: Yujin Powder
YJP-A: YJP alcohol extracts
SASP: Sulfasalazine
CMC-Na: Carboxymethylcellulose sodium
MLNs: Mesenteric lymph nodes
DAI: Disease activity index
MPO: Myeloperoxidase
HE: Haematoxylin and eosin
SDS-PAGE: SDS-polyacrylamide gel electrophoresis
PVDF: Polyvinylidene fluoride
PBS: Phosphate buffered saline
SEM: Standard error of mean
ANOVA: Analysis of variance
PCA: Principal component analysis
PCOA: Principal coordinate analysis
GC: Germinal center
