# Supplementary material for: Regulation of Yujin Powder alcoholic extracts on ILC3s-TD IgA-colonic mucosal flora axis of DSS-induced ulcerative colitis
Source: Front Microbiol. 2022 Oct 20;13:1039884. doi: 10.3389/fmicb.2022.1039884 (PMC9633017; doi:10.3389/fmicb.2022.1039884)
Supplement: Supplementary file 1 [file Data_Sheet_1.ZIP › Description.pdf]

1. The description of resulting graph in colonic mucosal flora.

1.1 Species Venn diagram analysis was performed using the OmicStudio tools at https://www.omicstudio.cn/tool and the full original source data was shown in Data sheet 6.xlsx.

1.2 The pictures of ACE, Chao1, Shannon and Simpson indices were plotted using GraphPad Prism version 6.0 and the full original source data was shown in Data sheet 7.xlsx.

1.3 The rank abundance curve was plotted using python's matplotlib (v3.3.4) and the full original source data was shown in Rank abundance curve.reabundance.

1.4 The rarefaction curve was plotted using python and the full original source data was shown in Data sheet 11.

1.5 The pictures of PCA and PCOA were plotted using R language tools and the full original source data was shown in PCA.axes and PCOA.axes, respectively.

1.6 Difference analysis at phylum and genus levels was performed using the OmicStudio tools at https://www.omicstudio.cn/tool and GraphPad Prism version 6.0, the full original source data was shown in Data sheet 8-10.xlsx.

1.7 The LEfSe analysis was performed using analysis tool at http://huttenhower.sph.harvard.edu/lefse/ and the full original source data was shown in LEfSe.rep.

1.8 The correlation analysis was performed using the OmicStudio tools at https://www.omicstudio.cn/tool by Pearson correlation and the full original source data was shown in Data sheet 12.
