# Supplementary material for: Regulation of Yujin Powder alcoholic extracts on ILC3s-TD IgA-colonic mucosal flora axis of DSS-induced ulcerative colitis
Source: Front Microbiol. 2022 Oct 20;13:1039884. doi: 10.3389/fmicb.2022.1039884 (PMC9633017; doi:10.3389/fmicb.2022.1039884)
Supplement: Supplementary file 1 [file Data_Sheet_1.ZIP › Presentation.pdf]

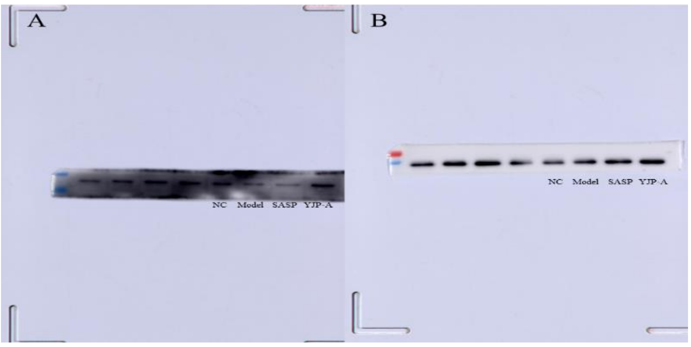

Figure S1. Whole un-cropped images of the original western blots A: MHC II, B: β-Tublin.

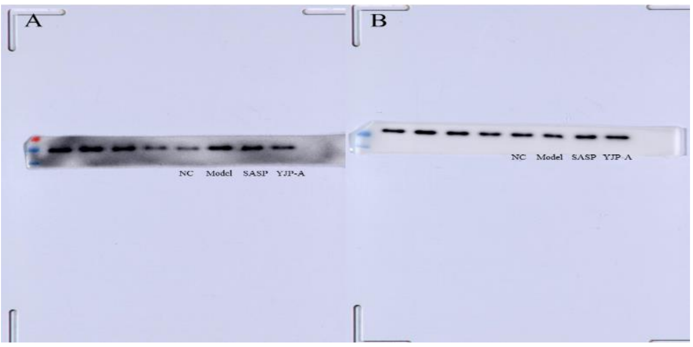

Figure S2. Whole un-cropped images of the original western blots A: Bcl6, B: GAPDH.

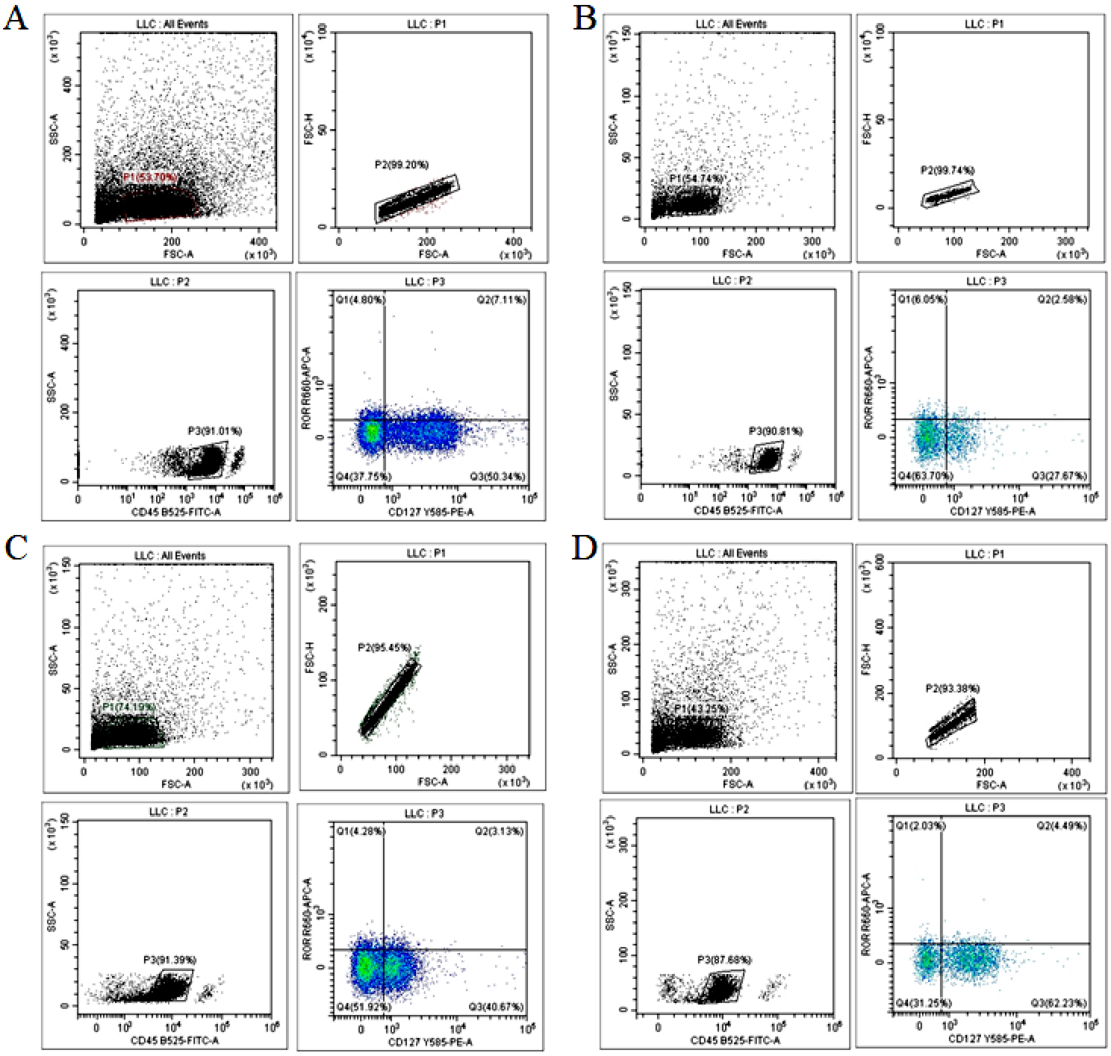

Figure S3. The gating strategies of ILC3s for flow cytometry. A: NC group; B: Model group; C: SASP group; D: YJP-A-H group.

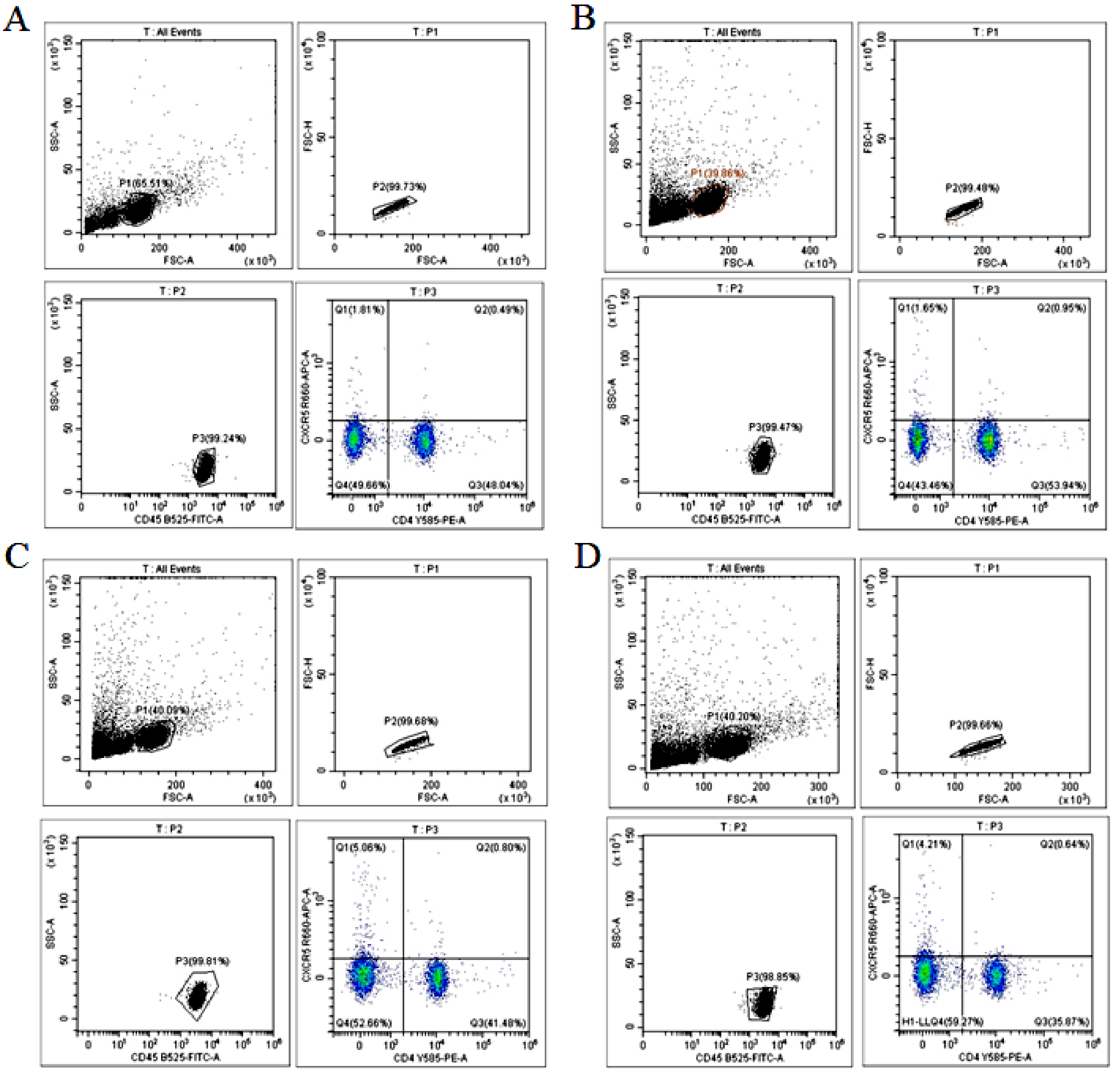

Figure S4. The gating strategies of Tfh cells for flow cytometry. A: NC group; B: Model group; C: SASP group; D: YJP-A-H group.

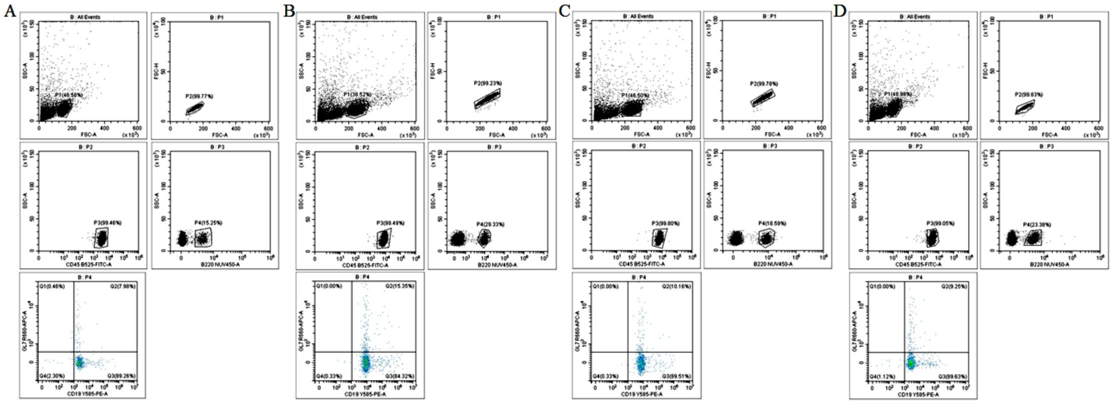

Figure S5. The gating strategies of B cells for flow cytometry. A: NC group; B: Model group; C: SASP group; D: YJP-A-H group.

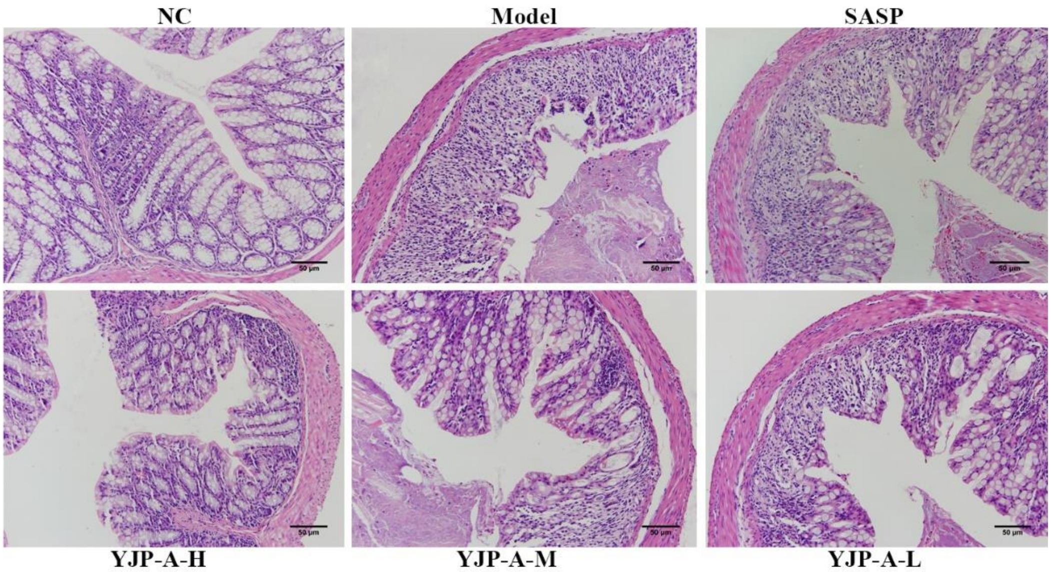

Figure S6. Raw figures of HE staining (200 × magnification).

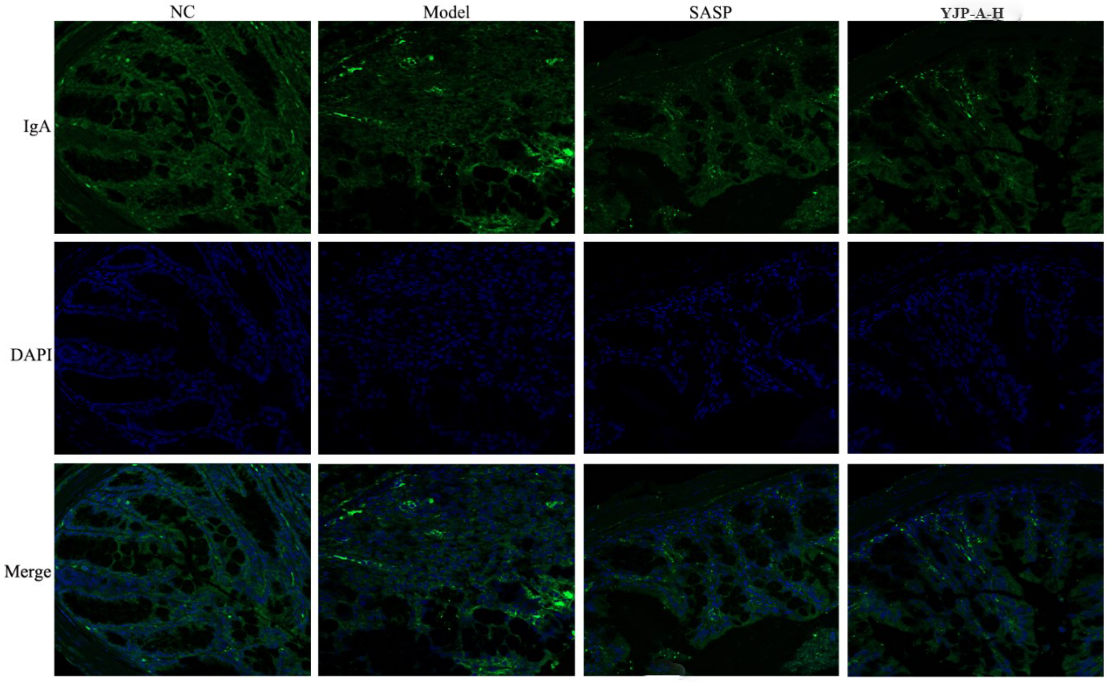

Figure S7. Raw figures of immunofluorescence assay (200 × magnification).
